# A Chronobiological Prognostic Model for Resected Stage I Non-Small-Cell Lung Cancer †

**DOI:** 10.3390/cancers18193208

**Published:** 2026-10-04

**Authors:** Giovanni Leuzzi, Pamela Minicozzi, Paolo Verderio, Chiara Maura Ciniselli, Michele Ferrari, Alessandro Pardolesi, Alessia Stanzi, Jury Brandolini, Luigi Rolli, Matteo Calderoni, Clarissa Uslenghi, Ugo Pastorino, Giuseppe Lo Russo, Claudia Proto, Arsela Prelaj, Teresa Beninato, Daniele Lorenzini, Piergiorgio Solli

**Affiliations:** 1 Division of Thoracic Surgery, Fondazione IRCCS Istituto Nazionale dei Tumori, 20133 Milan, Italy; michele.ferrari@istitutotumori.mi.it (M.F.); alessandro.pardolesi@istitutotumori.mi.it (A.P.); alessia.stanzi@istitutotumori.mi.it (A.S.); jury.brandolini@istitutotumori.mi.it (J.B.); luigi.rolli@istitutotumori.mi.it (L.R.); matteo.calderoni@istitutotumori.mi.it (M.C.); clarissa.uslenghi@istitutotumori.mi.it (C.U.); piergiorgio.solli@istitutotumori.mi.it (P.S.); 2 Unit of Bioinformatics and Biostatistics, Fondazione IRCCS Istituto Nazionale dei Tumori, 20133 Milan, Italy; paolo.verderio@istitutotumori.mi.it (P.V.); chiara.ciniselli@istitutotumori.mi.it (C.M.C.); 3 Fondazione IRCCS Istituto Nazionale dei Tumori, 20133 Milan, Italy; ugo.pastorino@me.com; 4 Department of Medical Oncology, Fondazione IRCCS Istituto Nazionale dei Tumori, 20133 Milan, Italy; giuseppe.lorusso@istitutotumori.mi.it (G.L.R.); claudia.proto@istitutotumori.mi.it (C.P.); arsela.prelaj@istitutotumori.mi.it (A.P.); teresa.beninato@istitutotumori.mi.it (T.B.); 5 Department of Diagnostic Innovation, Fondazione IRCCS Istituto Nazionale Dei Tumori, 20133 Milan, Italy; daniele.lorenzini@istitutotumori.mi.it; 6 Department of Oncology and Hemato-Oncology, University of Milan, 20133 Milan, Italy

**Keywords:** chronobiology, NSCLC, season, prognosis, circadian rhythm, inflammation

## Abstract

Objectives: Chronobiology may influence outcomes in non-small-cell lung cancer (NSCLC), but its prognostic role in early-stage disease remains unclear. We evaluated the association between perioperative chronobiological variables, systemic inflammation, and long-term outcomes after complete resection for stage I NSCLC. Methods: 587 patients who underwent curative-intent resection for pathological stage I NSCLC between 2009 and 2018 were retrospectively reviewed. Chronobiological and inflammatory variables included season of surgery (spring/summer [21st March–22nd September], autumn/winter [23rd September–20th March]), time of day, day of week, and preoperative (CRP0 levels: low ≤ 3 mg/L, high > 3 mg/L) and postoperative day-3 (CRP3 levels: low ≤ 126 mg/L, high > 126 mg/L) C-reactive protein levels. The cohort was randomly divided into training (70%) and testing (30%) sets to develop and validate a prognostic model for overall survival (OS), taking into consideration the follow-up time. Disease-free survival (DFS) was analyzed as a secondary endpoint. Results: Median follow-up was 6.7 years, with 80 deaths and 108 recurrences recorded. In the training set, older age (*p* = 0.0049), stage IB disease (*p* = 0.0301), high CRP0 (*p* = 0.0049), and high CRP3 (*p* = 0.0280) were associated with worse OS, while surgery performed during autumn/winter was associated with improved OS (*p* = 0.0312). The multivariate Cox model including age, stage, and season of surgery showed the best prognostic performance (C-index 0.67, 95% Bonferroni-adjusted CI 0.50–0.83) and was internally validated in the testing cohort (C-index 0.69, 95%CI 0.52–0.86). The resulting linear predictor remained associated with both OS and DFS in the overall population. Conclusions: A simple model integrating age, pathological stage, and season of surgery was associated with long-term outcomes after surgery in stage I NSCLC. This model provides an exploratory framework integrating host-, tumor-, and chronobiological dimensions of prognosis. These findings require external validation and further investigation of the biological mechanisms underlying seasonal variation.

## Simple Summary

This study examined whether the timing of lung cancer surgery may be associated with long-term outcomes. Among 587 patients with early-stage NSCLC, those undergoing surgery in autumn or winter showed better survival, while time of day and day of week were not associated with outcome. A simple model combining age, tumor stage, and season of surgery helped identify prognostic differences, suggesting that timing may reflect biological factors linked to survival.

## 1. Introduction

Surgical resection remains the standard treatment for stage I non-small-cell lung cancer (NSCLC). However, survival after surgery is still heterogeneous, even within the same anatomical stage. The TNM classification is the main prognostic tool, but it does not entirely capture host-related factors that may influence postoperative outcomes [1]. For this reason, additional variables reflecting the patient’s biological condition are increasingly being explored.

Systemic inflammation is one of the most studied host-related determinants. C-reactive protein (CRP), measured before or shortly after surgery, has been associated with long-term mortality in resectable NSCLC [2,3]. These results indicate that the patient’s inflammatory status may affect prognosis. However, CRP is measured at a single time point and might fail to capture the dynamic physiological processes that occur around surgery.

Chronobiology may provide complementary information. Circadian and seasonal rhythms regulate several physiological processes, including immune activity, hormonal balance, and response to stress [4,5]. In cancer, disruption of these rhythms has been linked to tumor progression and alterations in the tumor microenvironment (TME) [6,7,8,9,10]. In surgical settings, temporal variables such as time of day (ToD), day of week (DoW), and season of surgery may therefore influence postoperative outcomes.

Among these factors, season of surgery can be considered a wider temporal indicator, as it reflects environmental and biological variations over longer periods. Previous studies have suggested a possible association between season of diagnosis or operation and survival in lung cancer [11,12,13,14]. In addition, environmental aspects such as sunlight exposure and vitamin D levels have been linked to prognosis in early-stage NSCLC [15,16,17]. However, the mechanisms underlying these observations are still unclear. In particular, it is yet uncertain whether temporal effects are mediated by systemic inflammation or reflect additional biological pathways not captured by conventional biomarkers. In this context, CRP may represent a static snapshot of the inflammatory state, whereas the season of surgery may reflect a broader temporal biological context [18].

The aim of this study was to evaluate the independent and combined prognostic role of perioperative chronobiological factors and systemic inflammation in patients undergoing complete resection for stage I NSCLC. We also aimed to develop and internally validate a prognostic model based on clinically available variables.

## 2. Methods

### 2.1. Study Population

All consecutive patients who underwent curative-intent resection for stage I NSCLC at Fondazione IRCCS Istituto Nazionale dei Tumori between January 2009 and December 2018 were included in the present study. Patients were clinically followed up to April 2025 according to institutional protocols, with regular clinical and radiological assessments. All cases were retrospectively re-staged according to the 9th edition of the TNM classification [1] based on the available pathological and surgical records.

Inclusion criteria were pathological stage I disease, complete resection (R0), systematic lymphadenectomy, and availability of clinical, laboratory, and follow-up data. Patients with missing key variables, including C-reactive protein (CRP) measurements or follow-up information, were excluded.

The study was approved by the Institutional Review Board of Fondazione IRCCS Istituto Nazionale dei Tumori (Protocol No. INT 6-25, approval date: 7 February 2025) and was conducted in accordance with the Declaration of Helsinki. According to the Italian Privacy Code (D.Lgs. 196/2003, Art. 110-bis, paragraph 4) and considering the retrospective design of the study within the institutional research activity of IRCCS institutions, the Institutional Review Board authorized the use of anonymized clinical data and waived the requirement for individual informed consent. Data confidentiality and patient privacy were maintained throughout the study.

### 2.2. Variables

Information on patients’ age at surgery, inflammation status, tumor characteristics, and surgical data were retrieved from institutional clinical records.

Given the availability of complete date and time of surgery, year of surgery, and predefined chronobiological variables were retrieved, including season of surgery (spring/summer [21st March–22nd September] vs. autumn/winter [23rd September–20th March]), DoW (Monday/Tuesday, Wednesday, Thursday/Friday), and ToD of surgery.

Systemic inflammation was assessed using CRP levels. Preoperative CRP (CRP0) was defined as the most recent CRP level recorded before surgery, while postoperative CRP (CRP3) was defined as the CRP level measured on postoperative day 3. According to previous analyses from our group, thresholds of 3 mg/L and 126 mg/L were used to identify patients with low and high CRP0 and CRP3 levels, respectively [2].

### 2.3. Study Endpoints and Follow-Up

Overall survival (OS) and disease-free survival (DFS) were the primary and secondary endpoints, respectively. OS follow-up time was defined as the time from surgery to death from any cause, with censoring at the date of last follow-up. DFS follow-up time was defined as the time from surgery to disease recurrence, with censoring at the date of last follow-up.

### 2.4. Statistical Analysis

Continuous variables were summarized using medians and interquartile ranges (IQRs), while categorical variables were reported as frequencies and percentages.

To develop and validate the prognostic model, the study cohort was stratified according to OS follow-up time and randomly split into a training set, including 70% of patients, and a testing set, including the remaining 30%.

In the training set, the association between OS and clinical variables was explored using Kaplan–Meier curves with log-rank tests for categorical variables, fringe plots for continuous variables, and univariate and multivariable Cox proportional hazards models. The relationship between continuous variables and the outcome was further investigated using regression models based on restricted cubic splines [19].

Only statistically significant variables at univariate analysis were selected for multivariate analyses. Interactions among significant variables were investigated using bivariate Cox regression models, including the two main effects and their first-order interaction term. To prevent collinearity, pairwise associations among significant variables were investigated using chi-squared, Fisher’s exact, or Wilcoxon tests, according to the nature of the investigated variables.

The predictive capability of multivariate prognostic models was quantified using Harrell’s concordance index (C-index) and corresponding 95% confidence intervals (CIs) [20]. The linear combination of variables included in the multivariate model with the highest statistically significant C-index, defined as a nominal lower limit of 95% CI > 0.50, was adopted as the linear predictor. To account for multiple hypothesis testing, a Bonferroni-adjusted CI was calculated for each C-index.

The linear predictor thus derived from the training set was subsequently validated in the testing set using the coefficients estimated in the training cohort. Following validation, its prognostic role for both OS and DFS was further assessed in the overall study population. Patients were also stratified into tertiles according to the distribution of the linear predictor, and survival differences among groups were assessed using Kaplan–Meier analysis.

To investigate whether the linear predictor was dependent on secular changes occurring during the study period, a sensitivity analysis was implemented on the whole dataset by adjusting the validated multivariate model for the year of surgery. Furthermore, to provide an additional internal validation of all variables included in the linear predictor, we supplemented the analyses by using a bootstrap-based resampling technique. In particular, we implemented sensitivity analyses using a multivariate bootstrap variable selection procedure (boot.stepAIC function in R), which we used to evaluate predictors within a multivariable framework across 1000 bootstrap resamples. Within each bootstrap loop, multicollinearity was dynamically assessed using the Variance Inflation Factor. The final model inclusion frequency was calculated for each predictor across all bootstrap replicates, and variables with a selection frequency exceeding 70% were retained to implement multivariable Cox proportional hazards models on non-collinear variables.

Additional exploratory analyses investigated the association between CRP levels and the prognostic model, as well as the relationship between CRP and season of surgery.

Reporting of this study was assessed according to the TRIPOD statement [21,22], with the completed checklist provided in Supplementary Table S1.

All statistical analyses were performed using SAS Studio version 5.2 (SAS Institute, Cary, NC, USA) and R (version 4.6.1). A nominal two-sided *p*-value < 0.05 was considered statistically significant.

## 3. Results

### 3.1. Patients’ and Tumor Characteristics

Overall, 587 patients underwent curative-intent resection for stage I NSCLC (Table 1). Median age was 68 years (IQR 62–74). Most patients were men (81%), smokers (80%), and had chronic obstructive pulmonary disease (64%). Stage IA disease was present in 417 patients (71%), while 170 patients (29%) had stage IB disease. Lobectomy was performed in 470 patients (80%). Most patients underwent surgery on Thursday/Friday (285, 49%), with a median surgical start time of 10:00 a.m. Surgery was distributed across the year, with 278 patients (47%) treated in spring/summer and 309 (53%) in autumn/winter. Median OS follow-up was 6.7 years (IQR 4.7–9.0). Overall, 80 deaths and 108 relapses were recorded. After exclusion of patients with missing CRP or follow-up data, 554 patients were included in the score analysis and randomly assigned to the training (*n* = 388) and testing (*n* = 166) sets (Supplementary Figure S1).

### 3.2. Prognostic Score Development

**Figure 1:**
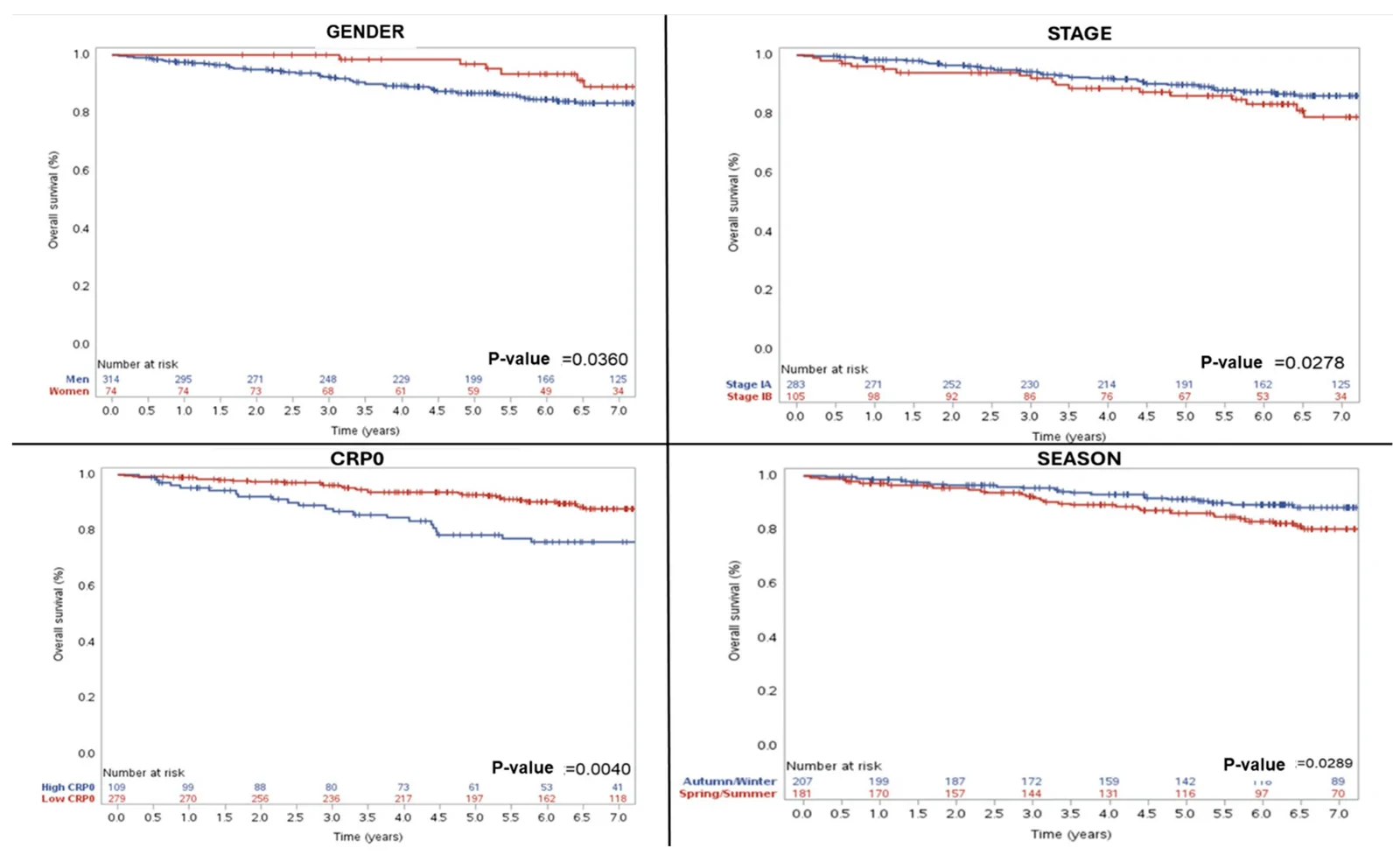
Kaplan–Meier survival curves for statistically significant variables in univariate analysis. CRP0, preoperative C-reactive protein (CRP).

In the training set, univariate Cox analysis showed that older age (hazard ratio [HR] 1.05, 95% CI 1.01–1.08, *p* = 0.0049), pathological stage IB (HR 1.80, 95% CI 1.06–3.06, *p* = 0.0301), high CRP0 (HR 2.12, 95% CI 1.26–3.57, *p* = 0.0049), and high CRP3 (HR 1.81, 95% CI 1.07–3.09, *p* = 0.0280) were associated with worse OS (Table 2). Being a woman (HR 0.42, 95% CI 0.18–0.97) and surgery performed in autumn/winter (HR 0.56, 95% CI 0.33–0.95, *p* = 0.0312) were associated with better OS. ToD and DoW were not associated with OS. Kaplan–Meier curves for significant variables are shown in Figure 1.

After evaluation of significant factors, interactions, and collinearity (Supplementary Table S2), five multivariate models were tested. The fully adjusted model (i.e., the primary model hereafter) including age (β coefficient = 0.040288), stage (β coefficient = 0.46048), and season of surgery (β coefficient = −0.50902) showed the best predictive capability (C-index 0.67, 95% Bonferroni-adjusted CI 0.50–0.83) and the lowest Akaike Information Criterion and was used to derive the linear predictor (Table 3, Supplementary Tables S3 and S4), although season was not independently significant (*p*-value = 0.06).

### 3.3. Prognostic Score Validation and Application

**Figure 2:**
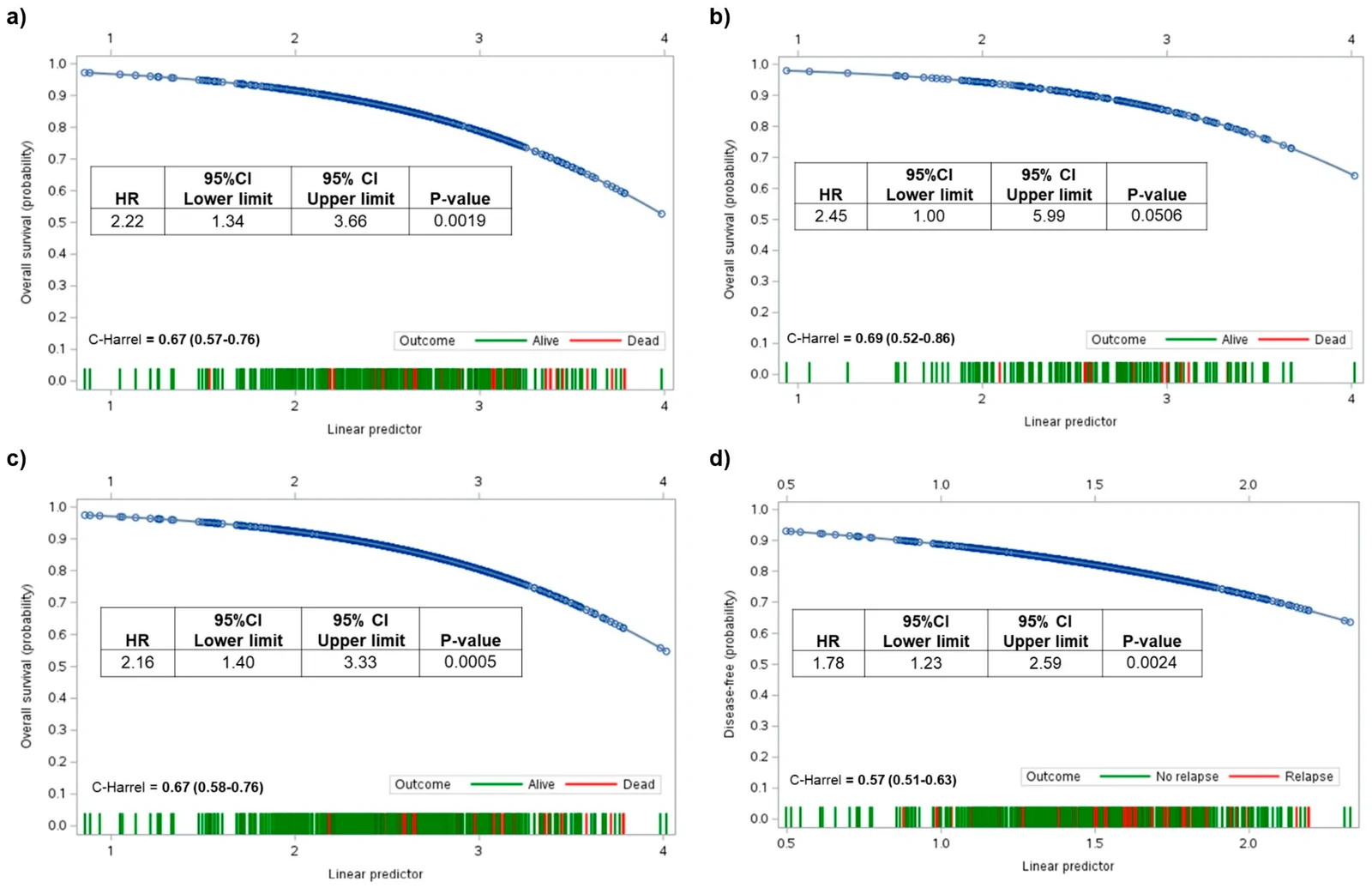
Survival fringe plots for the linear predictor in training (Panel (**a**)), testing (Panel (**b**)) and whole (Panels (**c**,**d**)) datasets. Survival estimated at median follow-up time. Blue line: linea predictor.

Figure 2a displays the estimated OS against the linear predictor for individual patients in the training set, categorized as alive (green ticks) or dead (red ticks). OS decreased as linear predictor values increased, with a more pronounced decline corresponding to higher mortality.

The linear predictor was validated in the testing set, where it remained associated with OS (HR 2.45, 95% CI 1.00–5.99) and showed a C-index of 0.69 (95% CI 0.52–0.86) (Figure 2b). In the whole cohort, the linear predictor was associated with OS (HR 2.16, 95% CI 1.40–3.33; C-index 0.67, 95% CI 0.58–0.76) and DFS (HR 1.78, 95% CI 1.23–2.59; C-index 0.57, 95% CI 0.51–0.63) (Figure 2c and Figure 2d, respectively).

**Figure 3:**
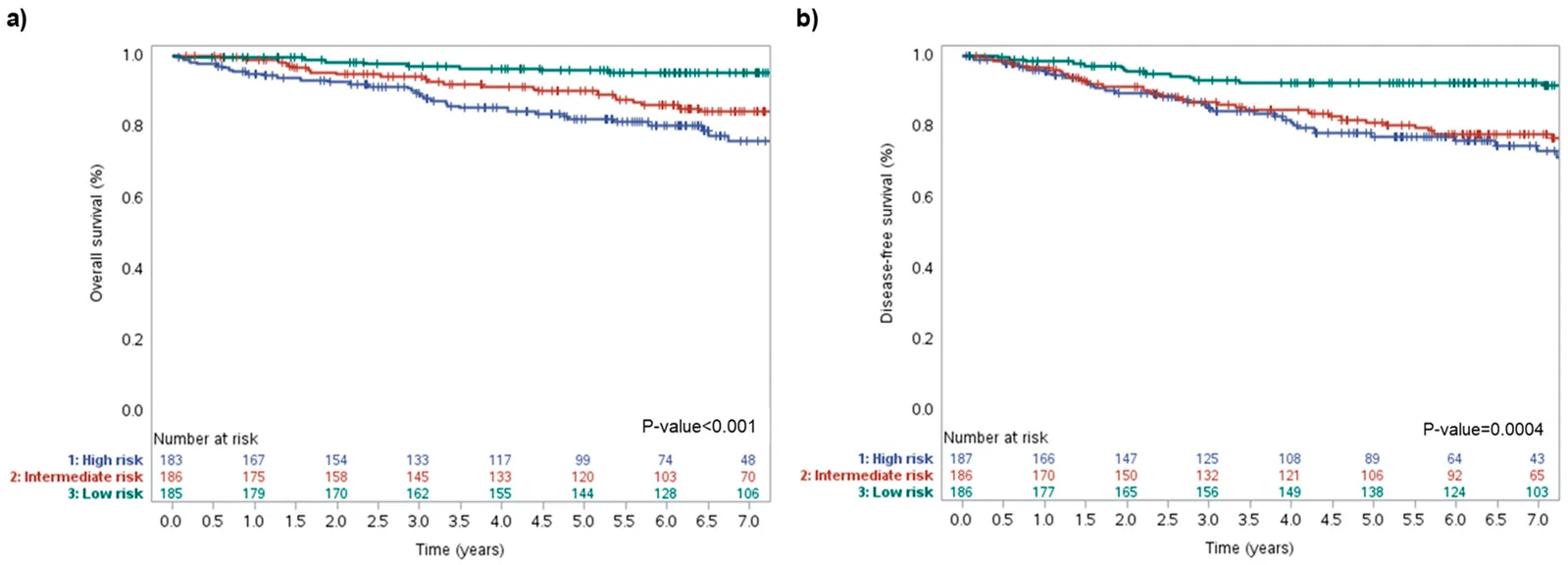
Kaplan–Meier curves for overall (Panel (**a**)) and disease-free (Panel (**b**)) survival according to linear predictor tertiles in the whole dataset.

For descriptive purposes, patients were stratified into three (low, intermediate and high) risk groups, according to tertiles of the overall distribution of the linear predictor (1st tertile = 2.364, 2nd tertile = 2.788, 3rd tertile = 4.017). OS and DFS differed significantly across risk groups (log-rank *p* < 0.001 and *p* = 0.0004, respectively; Figure 3). Exploratory analyses showed that the linear predictor was associated with CRP0 (*p* = 0.0161) and CRP3 (*p* = 0.0044), whereas CRP0 and CRP3 levels did not vary significantly according to season of surgery (*p* = 0.9822 and *p* = 0.2052, respectively; Supplementary Figure S2).

The sensitivity analysis on the whole dataset, which was implemented by adjusting for the year of surgery in the internally validated multivariate model, showed that including the year of surgery did not materially affect model discrimination. In fact, the age, stage, season and year of surgery model resulted in a comparable C-index of 0.68 (0.58–0.77) for OS and of 0.58 (0.52–0.64) for DFS.

Finally, the analyses carried out using a bootstrap-based resampling technique as further internal validation showed that age, CRP0 and season of surgery were selected with a selection frequency ranging from 78.8 to 90.9%, whereas stage was selected with a frequency of 51.8% and gender of 55.2% (Supplementary Table S5). Considering those variables selected with a frequency of at least 70% and accounting for the association between CRP0 and age (*p*-value = 0.0441), two bootstrap-based sensitivity models were implemented on the overall population. The model including age and season showed the highest predictive capability (C-index 0.63, 95% Bonferroni-adjusted CI 0.56–0.70), which overlapped with the one estimated for the age-only model (C-index 0.62, 95% Bonferroni-adjusted CI 0.54–0.70). As before, the adjustment of the age-season model for year of surgery did not affect its discrimination (C-index 0.66, 95% Bonferroni-adjusted CI 0.59–0.73). A different result was found for stage: adjusting the age-season model for it did not affect model discrimination on the overall population (C-index 0.64, 95% Bonferroni-adjusted CI 0.57–0.71), but it improved it in the training set, where C-index increased from 0.61 to 0.67 (Supplementary Table S4).

## 4. Discussion

This study evaluated the prognostic significance of perioperative chronobiological and inflammatory variables in a homogeneous cohort of patients undergoing complete resection for stage I NSCLC. In the multivariate analysis, the primary model including age, pathological stage, and season of surgery showed moderate prognostic discrimination and was internally assessed in the testing set. To note, inclusion of year of surgery did not materially affect model discrimination, with the four-variable age–stage–season-year of surgery model showing comparable performance. This suggests that the prognostic information captured by the model, including year of surgery, was not substantially dependent on secular changes occurring during the study period. CRP0 and CRP3 were associated with OS in univariate analysis, confirming the prognostic relevance of systemic inflammation in resectable NSCLC [2,3]. However, CRP was not retained in the multivariable prognostic model, whereas season of surgery remained part of the validated model. Overall, these findings support further investigation of season of surgery as a potential chronobiological dimension of prognosis, alongside host- and tumor-related factors.

The association between chronobiology and cancer progression has been increasingly investigated. Circadian rhythms regulate multiple physiological processes relevant to oncological control, including immune cell trafficking, endocrine signaling, DNA repair, and inflammatory responses [4,5]. In NSCLC, dysregulation of clock-related genes has been associated with tumor progression and TME composition. Xian et al. identified several clock-related genes, including TIMELESS and RORA, associated with survival and immune cell infiltration [6], while Yang et al. reported an association between circadian imbalance and an immunosuppressive TME phenotype [7]. These findings provide a biological rationale for investigating temporal organization in lung cancer.

In this context, season of surgery may be a clinically accessible surrogate for chronobiological exposure. Unlike circadian variables (which operate on daily cycles), season of surgery includes both environmental and biological variations over extended periods, such as photoperiod, sunlight exposure, hormonal oscillations, sleep modulation, and immune state. Previous studies have suggested an association between seasonality and survival after lung cancer surgery. For example, Turna et al. reported significantly improved survival among patients undergoing surgery during summer months [11]. Similarly, LaPar et al. reported seasonal variations in postoperative outcomes after lung cancer resection, thus indicating that ambient factors may influence perioperative recovery and long-term prognosis [23]. In Norway, Porojnicu et al. identified geographical and seasonal differences in lung cancer prognosis, likely related to sunlight exposure and vitamin D synthesis [24]. Noteworthy, the seasonal effect on OS seems heterogeneous across different regions. Specifically, cancer cohorts in Northern regions often experience a “summer benefit”; in contrast, Mediterranean studies have reported better outcomes during autumn or winter periods [18,25]. Rather than a direct effect of vitamin D alone, this paradox may reflect fluctuations in climate stress, sun exposure, and environmental “chronodisruption”. The potential role of vitamin D in NSCLC has also been investigated prospectively, although a randomized trial of vitamin D supplementation did not demonstrate a survival benefit in the overall study population [26].

The relationship between season of surgery and systemic inflammation should be interpreted cautiously. CRP is a well-established marker of systemic inflammation and has previously been associated with prognosis after lung cancer resection [2,3]. In our cohort, both CRP0 and CRP3 were associated with poorer OS in univariate analysis, but neither CRP0 nor CRP3 differed significantly according to season of surgery, while the linear predictor was associated with both CRP measures. These findings do not establish a mechanistic relationship between seasonality and systemic inflammation, but suggest that the observed seasonal signal is not simply explained by differences in perioperative CRP levels. From a biological perspective, CRP may represent a relatively transient measure of perioperative inflammatory activation, whereas season could reflect a broader temporal context encompassing immune, endocrine, metabolic, and environmental variations. Systemic inflammation might therefore represent only one component of a more complex temporal host condition. This interpretation remains hypothesis-generating and requires prospective biological validation.

Experimental and translational evidence further supports a relationship between circadian regulation and immune function. Circadian clocks influence lymphocyte trafficking and adaptive immune responses [27], while diurnal variation in CD8+ T-cell localization within the TME [28] and circadian disruption-related NF-κB activation and neutrophil recruitment [29] provide potential biological mechanisms linking temporal organization to antitumor immunity. These observations provide biological plausibility for a potential link between temporal organization and long-term oncological outcomes.

Time of day and day of week were not associated with survival in the present analysis. However, the absence of an observed association for time of day should be interpreted cautiously, as surgical start times were concentrated within a relatively narrow interval, with a median of 10:00 a.m. and an IQR of 9:00 a.m.–1:00 p.m., limiting the ability to detect potential circadian effects. Similarly, the day-of-week categories were defined to reflect the distribution of surgical activity across the working week and should be regarded as exploratory rather than biologically predefined groups. Therefore, these negative findings should not be interpreted as evidence against a possible circadian influence on surgical outcomes. Previous studies in systemic oncology have suggested that treatment timing may affect outcomes, particularly for immunotherapy [30,31]. However, surgical pathways are substantially more complex and may be influenced by anesthesia, operative complexity, perioperative care, and organizational factors. Further studies specifically designed to capture a wider circadian range and standardized perioperative exposures are needed to clarify whether time-dependent effects exist in thoracic surgery.

From a clinical perspective, these findings suggest that chronobiological variables may provide an additional dimension to conventional prognostic assessment in early-stage NSCLC. Notably, the age–stage–season primary model combines host-, tumor-, and chronobiological information using variables that are routinely available and do not require additional costs or molecular profiling. However, its discriminatory performance was only moderate, and the present findings should therefore be considered exploratory rather than immediately applicable to clinical decision-making. Importantly, the data do not support modifying surgical scheduling according to season. Rather, the observed associations provide a rationale for further prospective studies incorporating longitudinal immune profiling, circadian biomarkers, vitamin D assessment, and molecular clock analyses. Such studies may help determine whether chronobiological information can ultimately complement established clinicopathological prognostic factors in thoracic oncology [18].

### Study Limitations and Strengths

This study has several limitations. First, it has a retrospective single-center design, which introduces potential selection bias. Second, data on sunlight exposure, vitamin D, melatonin, and cortisol levels, immune cell fluctuations, and sleep habits were unavailable and should be assessed in future studies to confirm our interpretations. Postoperative systemic treatment was not included among the analyzed covariates, as adjuvant therapy during the study period was restricted to a limited proportion of selected patients with stage IB disease. Residual confounding related to unmeasured patient, organizational, or environmental factors associated with seasonality cannot be excluded. Third, chronobiological variables were categorized into fixed groups, which may oversimplify the underlying biology. Fourth, although the C-index of the primary age-stage-season model was 0.67 (0.54–0.79), its Bonferroni-adjusted CI was (0.50–0.83), thus limiting the discriminatory robustness of the model itself. Finally, given the exploratory nature of the analysis, the retrospective design, and the absence of external validation, the results should be interpreted with caution.

However, bootstrap-based models were also implemented on the whole dataset as an additional internal validation. The variables included and their predictive prognostic capability were comparable with those obtained by using the splitting technique, thus increasing our confidence in the internal consistency of the proposed prognostic primary model.

Despite these limitations, the strengths of this study include (i) a homogeneous cohort of completely resected stage I NSCLC patients, (ii) extended follow-up, (iii) consistent perioperative management, and (iv) internal validation of the prognostic model. Furthermore, the analyses of chronobiological and inflammatory variables offer a clinically pragmatic framework that may inform future translational research.

## 5. Conclusions

In conclusion, season of surgery was associated with long-term survival after complete resection for stage I NSCLC and was included, together with age and pathological stage, in a simple prognostic model with moderate discrimination. The age–stage–season primary model provides an exploratory and hypothesis-generating framework integrating host-, tumor-, and chronobiological dimensions of prognosis. Although the underlying biological mechanisms remain uncertain, these findings support further investigation of chronobiological factors in perioperative thoracic oncology and require external validation before clinical application.

## Figures and Tables

**Table 1:** Patients’ and tumor characteristics.

	All	
	*n*	%
Patients’ characteristics		
Age (years)	587	
Median (IQR)		68 (62–74)
Range (min-max)		(30–88)
Gender		
Men	478	81.43
Women	109	18.57
Smoker		
Yes	470	80.07
No	117	19.93
Previous tumor		
Yes	215	36.63
No	372	63.37
Chronic obstructive pulmonary disease		
Yes	376	64.05
No	211	35.95
Tumor characteristics		
Histology		
Squamous	102	17.38
Other	485	82.62
Lobe		
Superior	351	59.8
Hilar-Middle	25	4.26
Inferior	211	35.95
Laterality		
Right	362	61.67
Left	225	38.33
Stage		
IA	417	71.04
IB	170	28.96
Type of surgery		
Lobectomy	470	80.07
Other	117	19.93
Video-assisted thoracic surgery		
Yes	179	30.49
No	408	69.51
Year of surgery		
Median (IQR)	587	2015 (2012–2016)
Range (min-max)		(2009–2018)
Season of surgery		
Spring/summer	278	47.26
Autumn/winter	309	52.64
Day of surgery		
Monday/Tuesday	151	25.72
Wednesday	151	25.72
Thursday/Friday	285	48.55
Start of the surgery (hour)		
Median (IQR)	587	10 (9–13)
End of the surgery (hour)		
Median (IQR)	587	13 (11–15)
Length of the surgery (hour)		
Median (IQR)	587	2 (2–2)
Preoperative C-reactive protein (CRP0)		
Low	406	72.11 *
High	157	27.89 *
Missing	24	-
Postoperative C-reactive protein (CRP3)		
Low	427	72.87 *
High	159	27.13 *
Missing	1	-

IQR, interquartile range; * Percentages calculated after excluding patients with missing information on either CRP0 (24 out of 587 [4.09%]) or CRP3 (1 out of 587 [0.17%].

**Table 2:** Univariate Cox analysis (Hazard ratio [HR] and 95% confidence interval [CI]) for overall survival in the training set.

		95% CI		
	HR	Lower Limit	Upper Limit	*p*-Value
Patients’ characteristics				
Age (years, per unit increase)	1.05	1.01	1.08	0.0049
Gender				
Woman (vs. Man)	0.42	0.18	0.97	0.0422
Smoker				
Yes (vs. No)	1.36	0.67	2.76	0.4012
Previous tumor				
Yes (vs. No)	1.37	0.82	2.30	0.2330
Chronic obstructive pulmonary disease				
No (vs. Yes)	0.73	0.41	1.30	0.2900
Tumor characteristics				
Histology				
Other (vs. Squamous)	0.59	0.33	1.06	0.0773
Lobe				
Hilar-Middle (vs. Superior)	1.10	0.64	1.90	0.7202
Inferior (vs. Superior)	1.08	0.63	1.83	0.7875
Laterality				
Left (vs. Right)	0.87	0.50	1.50	0.6146
Stage				
IB (vs. IA)	1.80	1.06	3.06	0.0301
Type of surgery				
Other (vs. Lobectomy)	0.62	0.29	1.31	0.2081
Video-assisted thoracic surgery				
No (vs. Yes)	0.84	0.46	1.55	0.5823
Season of surgery				
Autumn/winter (vs. spring/summer)	0.56	0.33	0.95	0.0312
Day of surgery				
Wednesday (vs. Monday/Tuesday)	0.82	0.39	1.72	0.5906
Thursday-Friday (vs. Monday/Tuesday)	1.09	0.59	2.03	0.7855
Start of the surgery (hour, per unit increase)	0.99	0.90	1.09	0.9109
End of the surgery (hour, per unit increase)	1.01	0.92	1.11	0.8272
Length of the surgery (hour, per unit increase)	1.16	0.85	1.57	0.3507
Preoperative C-reactive protein (CRP0) level				
High (vs. Low)	2.12	1.26	3.57	0.0049
Postoperative C-reactive protein (CRP3) level				
High (vs. Low)	1.81	1.07	3.09	0.0280

**Table 3:** Multivariate Cox analysis (Hazard ratio [HR] and 95% confidence interval [CI]) for overall survival in the training set.

		95% CI		
	HR	Lower Limit	Upper Limit	*p*-Value
Age (years, per unit increase)	1.04	1.01	1.07	0.0097
Stage				
IB (vs. IA)	1.58	0.93	2.71	0.0931
Season of surgery				
Autumn/winter (vs. spring/summer)	0.60	0.35	1.02	0.0601

## Data Availability

The data underlying this article will be shared on reasonable request to the corresponding author, subject to institutional approval and applicable privacy regulations.

## Supplementary Materials

The following supporting information can be downloaded at: https://www.mdpi.com/article/10.3390/cancers18193208/s1, Figure S1: Flow chart of patients considered and included in the analyses; Figure S2: Association between season of surgery and preoperative (CRP0, Panel a) and post-operative (CRP3, Panel b) C-reactive protein (CRP) levels; Table S1: TRIPOD 2015 Adherence Checklist; Table S2: Pairwise associations between variables significant at univariate analysis for overall survival in the training set; Table S3: Multivariate Cox regression models, together with their corresponding predictive prognostic capability (Harrel’s C-index); Table S4: Multivariate Cox regression models, together with their corresponding predictive prognostic capability (Harrel’s C-index), by sequentially adding each candidate variable to the age-only baseline model; Table S5: Multivariate bootstrap variable selection frequency and calibration plot for the multivariate Cox proportional hazard model including non-collinear variables with a selection inclusion frequency of at least 70%.
