# Supplementary material for: A Chronobiological Prognostic Model for Resected Stage I Non-Small-Cell Lung Cancer
Source: Cancers (Basel). 2026 Oct 4;18(19):3208. doi: 10.3390/cancers18193208 (PMC13664035; doi:10.3390/cancers18193208)
Supplement: Supplementary file 1 [file cancers-18-03208-s001.zip › cancers-4471350-supplementary figures.pdf]

cancers

MDPI

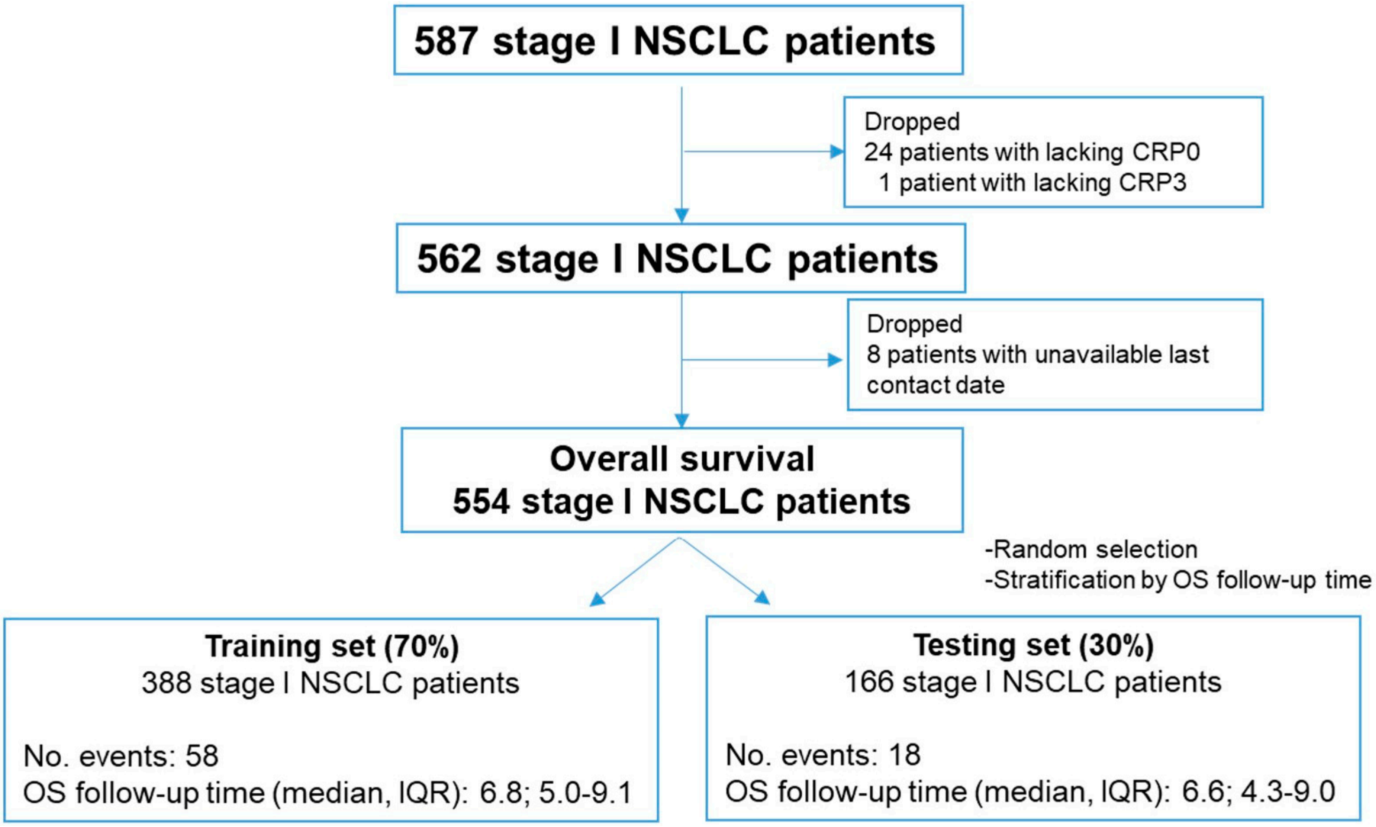

Supplementary Figure S1. Flow chart of patients considered and included in the analyses

CRP0, preoperative C-reactive protein (CRP); CRP3, postoperative CRP evaluated three days after surgery; IQR, interquantile range.

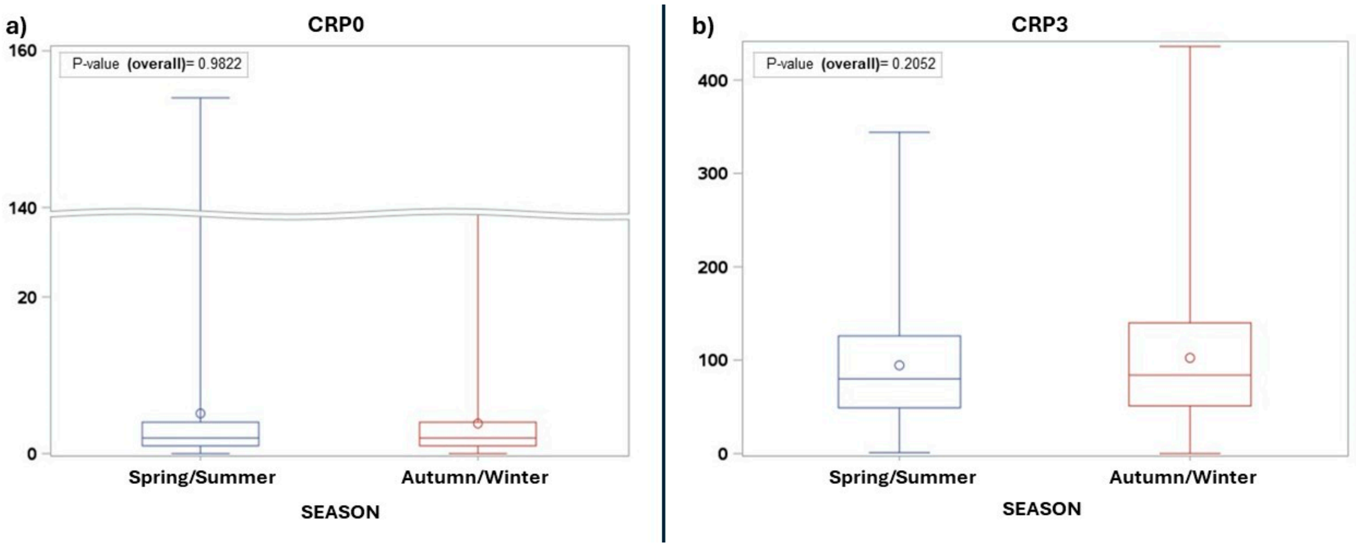

Supplementary Figure S2. Association between season of surgery and preoperative (CRP0, Panel a) and postoperative (CRP3, Panel b) C-reactive protein (CRP) levels.
