# Supplementary material for: A Chronobiological Prognostic Model for Resected Stage I Non-Small-Cell Lung Cancer
Source: Cancers (Basel). 2026 Oct 4;18(19):3208. doi: 10.3390/cancers18193208 (PMC13664035; doi:10.3390/cancers18193208)
Supplement: Supplementary file 1 [file cancers-18-03208-s001.zip › cancers-4471350-supplementary TABLE S5.pdf]

Supplementary Table S5. Multivariate bootstrap variable selection frequency and calibration plot for the multivariate Cox proportional hazard model including non-collinear variables with a selection inclusion frequency of at least 70%

	**Selection inclusion frequency (%)**	**Calibration plot* of the bootstrap selected multivariate model: Age + Season of surgery at median follow-up time (7 years)**
Age	90.9	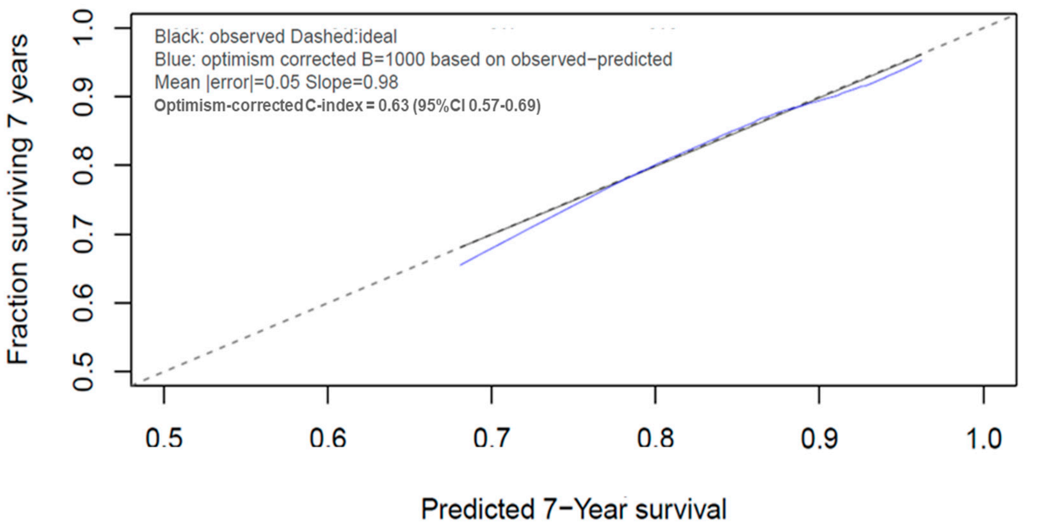
Preoperative C-reactive protein (CRP0)	90.1	
Season of surgery	78.8	
Previous tumour	62.7	
Gender	55.2	
Stage	51.8	
Chronic obstructive pulmonary disease	49.4	
Type of surgery	45.5	
Laterality	39.0	
Smoker	27.7	
Histology	24.8	
Lobe	23.5	
Day of surgery	22.6	
Video-assisted thoracic surgery	19.6	
Start of the surgery	17.8	

* A calibration plot evaluates how well the model's predicted probabilities match the actual observed outcome. Thus, model calibration was graphically assessed by plotting predicted survival probabilities against actual observed outcome using Kaplan-Meier estimator. To check the accuracy of the model and evaluate model overfitting, internal validation was performed using a bootstrap resampling procedure with 1000 replications (B=1000). The resulting calibration curve displays the bias-corrected estimates: if this curve lies exactly on this diagonal, it means the model is 100% accurate.
