# Supplementary material for: A Chronobiological Prognostic Model for Resected Stage I Non-Small-Cell Lung Cancer
Source: Cancers (Basel). 2026 Oct 4;18(19):3208. doi: 10.3390/cancers18193208 (PMC13664035; doi:10.3390/cancers18193208)
Supplement: Supplementary file 1 [file cancers-18-03208-s001.zip › Suppl Table S1 TRIPOD final.pdf]

Table S1 TRIPOD 2015 Adherence Checklist

Prediction model development and internal validation – Stage I NSCLC chronobiology study

Checklist requirements are paraphrased from the official TRIPOD 2015 development-and-validation checklist. Locations refer to the current revised manuscript. “Partial” identifies reporting that is present but not fully explicit.

Section / topic	Item	Type	Paraphrased TRIPOD requirement	Location in revised manuscript	Status / comment
Title and abstract	1	D;V	Identify the report as a multivariable prediction-model study and state target population/outcome.	p. 1, Title	Reported
Title and abstract	2	D;V	Summarize objectives, design, setting, participants/sample size, predictors, outcome, analysis, results, and conclusions.	pp. 1–2, Abstract	Reported
Introduction	3a	D;V	Describe the prognostic context and rationale, including relevant previous evidence/models.	p. 2, Introduction	Reported
Introduction	3b	D;V	State whether the objectives concern model development, validation, or both.	p. 2, end of Introduction	Reported
Methods – Source of data	4a	D;V	Describe study design/data source and distinguish development and validation data where applicable.	p. 3, §2.1; p. 4, §2.4	Reported
Methods – Source of data	4b	D;V	Give key dates: accrual period and end of follow-up.	p. 3, §2.1	Reported
Methods – Participants	5a	D;V	Describe clinical setting, number of centres, and location.	p. 3, §2.1	Reported
Methods – Participants	5b	D;V	Specify eligibility criteria.	p. 3, §2.1	Reported
Methods – Participants	5c	D;V	Describe relevant treatments received.	pp. 5–6, Table 1 (surgical procedure/VATS)	Partial – postoperative systemic treatment was not included among the analyzed covariates, as adjuvant therapy was restricted to a limited proportion of selected patients with stage IB disease.
Methods – Outcome	6a	D;V	Define predicted outcome(s), including timing and assessment.	p. 3, §2.3	Reported
Methods – Outcome	6b	D;V	Describe blinding of outcome assessment, if relevant.	—	N/A – retrospective outcome ascertainment
Methods – Predictors	7a	D;V	Define predictors and how/when they were measured.	p. 3, §2.2	Reported
Methods – Predictors	7b	D;V	Describe blinding of predictor assessment to outcome/other predictors, if relevant.	—	N/A – retrospective data extraction
Methods – Sample size	8	D;V	Explain how study size was determined.	p. 3, §2.1 (all consecutive eligible patients, 2009–2018)	Reported – no formal a priori sample-size calculation
Methods – Missing data	9	D;V	Describe how missing data were handled.	p. 3, §2.1; p. 5, §3.1; Table 1 footnote	Reported – complete-case exclusion for score analysis
Methods – Statistical analysis	10a	D	Describe how predictors were coded/handled analytically.	pp. 3–4, §§2.2 and 2.4	Reported
Methods – Statistical analysis	10b	D	Specify model type, model-building/selection procedures, and internal validation.	p. 4, §2.4	Reported
Methods – Statistical analysis	10c	V	For validation, explain how predictions/linear predictor were generated.	p. 4, §2.4 (training coefficients applied to testing set)	Reported
Methods – Statistical analysis	10d	D;V	State model-performance measures and methods used to compare models.	p. 4, §2.4 (Harrell C-index, 95% CI, Bonferroni-adjusted CI; AIC in Results/Supplement)	Reported
Methods – Statistical analysis	10e	V	Describe model updating/recalibration, if performed.	—	N/A – no model updating
Methods – Risk groups	11	D;V	Explain how risk groups were created, if used.	p. 4, §2.4; p. 12, §3.3	Reported – tertiles of the linear predictor
Methods – Development vs validation	12	V	For validation, report relevant differences between development and validation data.	p. 4, §2.4	N/A/limited – internal random split from the same source cohort
Results – Participants	13a	D;V	Report participant flow, outcome events, and follow-up; flow diagram if useful.	p. 5, §3.1; Supplementary Figure S1	Reported
Results – Participants	13b	D;V	Describe participant characteristics and missing predictor/outcome data.	pp. 5–6, Table 1	Reported
Results – Participants	13c	V	Compare key variable distributions between development and validation samples.	—	Partial – no separate train/test characteristics table; both samples derive from the same randomized cohort
Results – Model development	14a	D	Give numbers of participants and outcome events in each model analysis.	p. 5, §3.1 (n=554; training n=388; testing n=166)	Partial – split-specific death counts are not explicitly stated
Results – Model development	14b	D	Report unadjusted predictor–outcome associations, if assessed.	pp. 6–7, §3.2 and Table 2	Reported
Results – Model specification	15a	D	Provide the model specification needed for application, including regression coefficients and baseline information where required.	p. 9, §3.2 and Table 3	Partial – β coefficients are reported; model is used as a linear predictor and baseline

Section / topic	Item	Type	Paraphrased TRIPOD requirement	Location in revised manuscript	Status / comment
					survival is not reported
Results – Model specification	15b	D	Explain how the model/score is used.	pp. 9–12, §3.3 (linear predictor and tertile risk groups)	Reported
Results – Model performance	16	D;V	Report model-performance estimates with uncertainty.	pp. 9–12, §3.3; Figure 2; Supplementary Tables S3–S5	Reported
Results – Model updating	17	V	Report results of model updating, if done.	—	N/A – no model updating
Discussion – Limitations	18	D;V	Discuss important limitations, including sample, events, missing data, design, and validation limitations.	p. 15, Study Limitations and Strengths	Reported
Discussion – Interpretation	19a	V	Interpret validation results in relation to development performance.	pp. 12–15, Discussion	Reported
Discussion – Interpretation	19b	D;V	Provide an overall interpretation considering objectives, limitations, and existing evidence.	pp. 12–15, Discussion	Reported
Discussion – Implications	20	D;V	Discuss possible clinical implications and future research without overstating applicability.	pp. 14–16, Discussion and Conclusions	Reported
Other information – Supplementary resources	21	D;V	State availability of supplementary material, protocol/calculator/data where relevant.	Supplementary material; p. 16, Data Availability Statement	Reported
Other information – Funding	22	D;V	Report funding source and role of funder.	p. 16, Funding	Reported – source stated; funder role not separately described

D = model development; V = model validation. This manuscript develops a prognostic Cox model and evaluates it using an internal train/test split, with additional bootstrap-based sensitivity analyses.
