# Supplementary material for: A Chronobiological Prognostic Model for Resected Stage I Non-Small-Cell Lung Cancer
Source: Cancers (Basel). 2026 Oct 4;18(19):3208. doi: 10.3390/cancers18193208 (PMC13664035; doi:10.3390/cancers18193208)
Supplement: Supplementary file 1 [file cancers-18-03208-s001.zip › suppl table S2_270826.pdf]

Supplementary Table S2. Pairwise associations between variables significant at univariate analysis for overall survival in the training set

	**Age at surgery**	**Sex**	**Stage**	**CRP0**	**CRP3**	**Season of surgery**	**Year of surgery**
**Age at surgery**	—	0.0049		0.0398	0.0049		
**Sex**		—			0.0016		
**Stage**			—				
**Preoperative C-reactive protein (CRP0)**				—	<0.001		
**Postoperative C-reactive protein (CRP3) level**					—	0.0288	
**Season of surgery**						—	
**Year of surgery**							—

Chi-squared or Fisher's exact tests were used for categorical variables, whereas Wilcoxon tests were used for associations involving age at surgery and year of surgery. Empty cells indicate non-significant associations (p>0.05).
