# Supplementary material for: A Chronobiological Prognostic Model for Resected Stage I Non-Small-Cell Lung Cancer
Source: Cancers (Basel). 2026 Oct 4;18(19):3208. doi: 10.3390/cancers18193208 (PMC13664035; doi:10.3390/cancers18193208)
Supplement: Supplementary file 1 [file cancers-18-03208-s001.zip › suppl table S3_270826NoYearSurg.pdf]

Supplementary Table S3. Multivariate Cox regression models, together with their corresponding predictive prognostic capability (Harrel's C-index)

Model	Covariate	HR	95% CI Lower limit	95% CI Upper limit	P-value	C-index	95% Bonferroni-adjusted CI Lower limit	95% Bonferroni-adjusted CI Upper Limit	AIC*
Model 1						0.55	0.40	0.70	629.63
	Season of surgery (Autumn/winter vs spring/summer)	0.56	0.33	0.95	0.0312				
Model 2						0.61	0.44	0.78	623.67
	Preoperative C-reactive protein (CRP0; High vs Low)	1.99	1.17	3.36	0.0104				
	Season of surgery (Autumn/winter vs spring/summer)	0.61	0.36	1.03	0.0642				
	Stage (IB vs IA)	1.64	0.96	2.79	0.0714				
Model 3						0.67	0.50	0.83	622.71
	Age (per unit-increase)	1.04	1.01	1.07	0.0097				
	Season of surgery (Autumn/winter vs spring/summer)	0.60	0.35	1.02	0.0601				
	Stage (IB vs IA)	1.58	0.93	2.71	0.0931				
Model 4						0.64	0.50	0.78	625.52
	Sex (Female vs Male)	0.44	0.19	1.03	0.0591				
	Season of surgery (Autumn/winter vs spring/summer)	0.59	0.35	1.00	0.0496				
	Stage (IB vs IA)	1.65	0.97	2.81	0.0663				
Model 5						0.65	0.49	0.81	629.71
	Sex (Female vs Male)	0.45	0.19	1.05	0.0644				
	CRP0 (High vs Low)	1.96	1.16	3.32	0.0117				
	Season of surgery (Autumn/winter vs spring/summer)	0.60	0.36	1.02	0.0615				
	Stage (IB vs IA)	1.57	0.92	2.68	0.0993				

HR, hazard ratio; 95% CI, 95% confidence interval; *Akaike Information Criterion
