# Supplementary material for: A Chronobiological Prognostic Model for Resected Stage I Non-Small-Cell Lung Cancer
Source: Cancers (Basel). 2026 Oct 4;18(19):3208. doi: 10.3390/cancers18193208 (PMC13664035; doi:10.3390/cancers18193208)
Supplement: Supplementary file 1 [file cancers-18-03208-s001.zip › suppl table S4_270826_NoYearSurg_ccmpv.pdf]

Supplementary Table S4. Multivariate Cox regression models, together with their corresponding predictive prognostic capability (Harrel's C-index), by sequentially adding each candidate variable to the age-only baseline model

Incremental step	Covariate	HR	95% CI Lower limit	95% CI Upper limit	P-value	C-index	95% CI Lower limit	95% CI Upper Limit
Step 0						0.60	0.46	0.74
	Age (per unit-increase)	1.05	1.01	1.08	0.0049			
Step 1						0.61	0.48	0.75
	Age (per unit-increase)	1.04	1.01	1.08	0.0060			
	Season of surgery (Autumn/winter vs spring/summer)	0.57	0.34	0.97	0.0385			
Step 2						0.67	0.54	0.79
	Age (per unit-increase)	1.04	1.01	1.07	0.0097			
	Season of surgery (Autumn/winter vs spring/summer)	0.60	0.35	1.02	0.0601			
	Stage (IB vs IA)	1.58	0.93	2.71	0.0931			
